# Relationship between the rs2596542 polymorphism in the *MICA* gene promoter and HBV/HCV infection-induced hepatocellular carcinoma: a meta-analysis

**DOI:** 10.1186/s12881-019-0871-2

**Published:** 2019-08-16

**Authors:** Xiaojun Luo, Yu Wang, Ai Shen, Hejun Deng, Min Ye

**Affiliations:** Chongqing Key Laboratory of Translational Research for Cancer Metastasis and Individualized Treatment, Chongqing University Cancer Hospital & Chongqing Cancer Institute & Chongqing Cancer Hospital, No. 181 Hanyu Road, Shapingba District, Chongqing, 400030 China

**Keywords:** SNP, Major histocompatibility complex class I-related gene a (MICA), HBV/HCV-induced hepatocellular carcinoma (HCC), Hepatitis B virus (HBV), Hepatitis C virus (HCV), Meta-analysis

## Abstract

**Background & aims:**

Various studies have investigated the relationship between the polymorphism, rs2596542, in the promoter of the major histocompatibility complex class I-related gene A (*MICA*) gene with susceptibility to hepatitis B virus (HBV)/ hepatitis C virus (HCV)-induced hepatocellular carcinoma (HCC); however, the results are inconclusive. This meta-analysis was conducted to investigate the relationship between rs2596542 and HCV/HBV-induced HCC.

**Methods:**

Three electronic scientific publication databases (MEDLINE, Web of Science, and Embase) were screened using specific search terms and relevant literature identified using literature traceability methods. Selected publications were evaluated according to the inclusion and exclusion criteria, and 11 articles were included in the study. Effect size information (odds ratio [OR] and corresponding 95% confidence interval [CI]) were obtained following quality assessment and data extraction from the included publications, and a meta-analysis conducted.

**Results:**

A total of 11 publications were included in the study, including 4582 patients with HCC and 21,095 non-HCC patients. TT genotype at rs2596542 was a risk factor for the development of HCC in patients with HCV/HBV infection (OR = 1.248, 95% CI: 1.040–1.499, *P* = 0.017), particularly those with HCV infection (OR = 1.326, 95% CI: 1.101–1.599, *P* = 0.003) and Asians (OR = 1.273, 95% CI: 1.002–1.618, *P* = 0.048), or when the control group was patients with chronic hepatitis C (CHC) (OR = 1.506, 95% CI: 1.172–1.936, *P* = 0.001).

**Conclusion:**

The findings of this meta-analysis suggest that the rs2596542 variant in the *MICA* promoter region may affect MICA and soluble MICA (sMICA) protein expression, thereby influencing physiological vulnerability to HCC cells and the development of HCC. These data provide a theoretical basis for the diagnosis and treatment of patients with HCC and viral hepatitis infection.

**Electronic supplementary material:**

The online version of this article (10.1186/s12881-019-0871-2) contains supplementary material, which is available to authorized users.

## Background

Liver cancer is a common malignancy, and its mortality rate ranks third among malignant tumors; hence, this disease represents a serious threat to health and life [1]. Cirrhosis, caused by persistent hepatitis C virus (HCV) infection, is a key factor in the development of hepatocellular carcinoma (HCC) [2–4], while chronic hepatitis B virus (HBV) carriers are 100 times more likely to develop HCC than non-carriers [5–7]. HCV infection is a common cause of HCC in western countries and Japan, while HBV frequently causes HCC in other parts of Asia and developing countries [8].

Major histocompatibility complex class I-related gene A (*MICA*) is a tumor-associated gene containing numerous polymorphisms, which maps to the short arm of human chromosome 6 [9]. MICA is highly expressed in tumor cell lines and epithelial-derived primary tumors, such as lung, breast, liver, and prostate cancers [10]. MICA protein is a ligand of the NK cell surface activating receptor, NKG2D, which can effectively mediate NK cell killing of tumor cells by binding activation proteins [11]; however, many MICA-positive tumors release soluble MICA (sMICA) into the serum, which inhibits NK cell function [12]. *MICA* polymorphisms are associated with the development of numerous diseases, including cancer and autoimmune diseases [13, 14].

The rs2596542 single nucleotide polymorphism (SNP) in the *MICA* promoter region may be associated with HCC induced by HCV [15], while the results of studies investigating the association of rs2569542 with susceptibility to HBV/HCV-induced HCC are variable [16–18], limiting their credibility. The aim of this study was to perform a meta-analysis of published reports concerning rs2596542 and HCC susceptibility, to provide more reliable evidence for basic research and clinical treatment.

## Methods

### Search strategy

A comprehensive search of three databases (MEDLINE, Web of Science, and Embase) was performed and relevant publications were retrieved by literature traceability, from inception to April 2019. The search phrase used was: (“liver cell carcinoma” or “carcinoma, hepatocellular” or “Hepatocellular carcinoma” or HCC or hepatoma) AND (MICA or “MHC class I polypeptide-related sequence A” or “Human Major Histocompatibility Complex class I polypeptide-related sequence A”) AND (“polymorphism” or “SNP” or “variation” or “variants” or “locus” or “mutation”).

### Literature selection

All retrieved publications were screened by stepwise assessment of the title, abstract, and full text, according to the preset inclusion and exclusion criteria described below. Two independent investigators (Luo and Wang) conducted this work simultaneously, and any disagreement was resolved by discussion with a third investigator.

Inclusion criteria: (1) Studies including rs2596542 genotype and allele frequency data from patients with virus-induced live cirrhosis (LC), chronic hepatitis C (CHC)/chronic hepatitis B (CHB), and HCC; (2) Case-control studies; (3) Sufficient data to calculate an odds ratio (OR) and corresponding 95% confidence interval (CI); (4) In repeatedly published studies, the report with higher quality data and more comprehensive outcomes data, was selected; (5) English language.

Exclusion criteria: (1) Letters, notes of meetings, reviews, etc.; (2) Data could not be extracted.

### Quality assessment of the studies and data extraction

Quality assessment of the studies and data extraction were performed independently by two investigators (Shen and Deng), based on a set of predetermined criteria (Table 1) extracted and modified from previous studies [19–23]. Any disagreement was resolved by discussion with a third investigator. Quality scores ranged from 0 to 10, with higher scores representing better quality. The following information was extracted: first author, year of publication, country, the characteristics of participants in each study, sample size, number of cases, number of controls, sample source, detection method, and Hardy-Weinberg equilibrium (HWE) in controls.

**Table 1:** Scale for methodological quality assessment

Criteria	Score
1.Representativeness of cases	
1.1 HCC and LC diagnosed according to acknowledged criteria.	2
1.2 Mentioned the diagnosed criteria but not specifically described.	1
1.3 Not Mentioned.	0
2.Source of controls	
2.1 Cases and control from the same cohort	1
2.1.1 Defined HCC-free controls according to clinical, biochemical and serological parameters	2
2.1.2 Only defined HCC-free controls as CHB or LC but not described	1
2.2 Not described	0
3.Sample size	
3.1 > 300	2
3.2100–300	1
3.3 < 100	0
4.Quality control of genotyping methods	
4.1 Repetition of partial/total tested samples with a different method	2
4.2 Repetition of partial/total tested samples with the same method	1
4.3 Not described	0
5.Hardy-Weinberg equilibrium (HWE)	
5.1 Hardy-Weinberg equilibrium in control subjects	1
5.2 Hardy-Weinberg disequilibrium in control subjects	0

### Statistical analysis

The chi-square test was used to assess HWE in control populations, with *P* > 0.05 considered to indicate consistency with HWE. Meta-analysis was performed using SAS 9.2 (SAS Institute Inc., Cary, NC, USA) and STATA 12.0 (STATA Corp, College Station, TX, USA) software. Pooled OR and 95% CI values were calculated to evaluate the association of *MICA* rs2596542 with susceptibility to HBV/HCV-induced HCC, based on five genetic models: 1) allelic, C vs. T; 2) heterozygous, CT vs. CC; 3) homozygote, TT vs. CC; 4) dominant, TT/CT vs. CC; and 5) recessive, TT vs. CC/CT. When heterogeneity existed among different studies (*P* value of Q test < 0.10 and/or I^2^ > 50%), pooled OR values were calculated using a random effect model, while a fixed effect model was used when heterogeneity analysis indicated *P* > 0.10 and/or I^2^ < 50%. Publication bias was checked using Egger’s test, and Begg’s linear regression with funnel plots, and was considered present when *P* < 0.05, or the funnel plot was asymmetrical [24].

## Results

### Study identification and selection

**Fig. 1:**
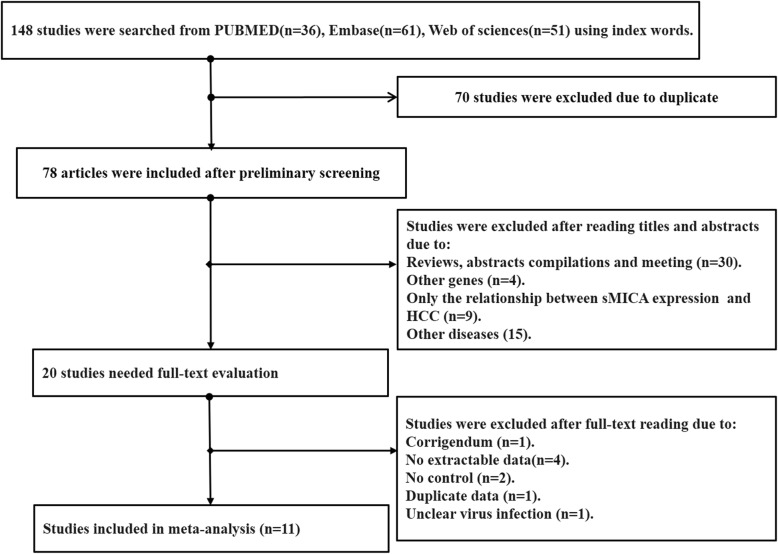
Flow diagram of the literature search and selection process

A total of 148 documents were retrieved from PubMed (*n* = 36), Embase (*n* = 61), and Web of Science (*n* = 51) databases, of which 70 were eliminated, due to duplicate publication. The remaining 78 articles were initially screened, based on the title and abstract, and 59 articles excluded for other reasons, including that: they were reviews, abstract compilations, or meeting reports (*n* = 30); they investigated other genes (*n* = 4); they only reported the relationship between sMICA expression and HCC (*n* = 9); or they investigated other diseases (*n* = 15). Finally, the full text documents of the remaining 20 articles were read, following which, nine were eliminated because they contained unsuitable data, such as corrigendum (*n* = 1), no extractable data (*n* = 4), no controls (*n* = 2), duplicate data (n = 1), and unclear virus infection (*n* = 1). Finally, 11 eligible publications [15–18, 25–31] were included in the meta-analysis (Fig. 1).

### General characteristics of the included studies

The 11 articles included in the study involved 25,677 blood samples from 4582 patients with HCC and 21,095 non-HCC patients. The subjects included Chinese, Japanese, Egyptian, Swiss, Vietnamese, and Italian individuals infected with HCV or HBV. Quality scores were > 6, indicating that this meta-analysis included high quality studies. SNP data from the control groups of the 11 studies were consistent with HWE. The general characteristics of the included studies are presented in Table 2.

**Table 2:** General characteristics description of the included studies

Study (first author/year)	Country	Enrolled population	Anti-HCV	Anti-HBV	Anti-HIV	Case	No-HCC	No.(HCC/no-HCC)	Sample source	Method	HWE	Score
Amal A. Mohamed/2017	Egypt	HCV-infected patients	positive	negative	negative	HCC	LC, healthy	47/94	blood samples	TaqMan ViiA 7 Real Time PCR System	Yes	6
Chung-Feng Huang/2017	China	HCV-infected patients	positive	negative	negative	HCC	LC, HCV patients	58/647	blood samples	ABI TaqMan® SNP genotyping assays	Yes	7
H. V. Tong/2013	Vietnam	HBV-infected patients	negative	positive	negative	HCC	LC, CHB, ASYM, non-HBV healthy	163/776	blood samples	asymmetrical PCR	Yes	9
Hoang Hai/2017	Japan	HCV-infected patients	positive	–	–	HCC	no-HCC	257/1098	blood samples	TaqMan SNP Genotyping Assays and direct sequencing	Yes	8
Kangmei Chen/2013	China	HBV-infected patients	negative	positive	negative	HCC	CHB	506/772	blood samples	TaqMan assays and 5% samples were randomly selected and directly sequenced	Yes	9
Maria Antonella Burza/2016	Italy	HCV-infected patients	positive	negative	negative	HCC	LC, no/mild fibrosis	142/311	blood samples	TaqMan assays	Yes	8
Paulisally Hau Yi Lo /2013	Japan	HCV-infected patients	positive	negative	negative	HCC	LC, CHC	1394/1629	blood samples	Illumina HumanHap610-Quad BeadChip or invader assay	Yes	9
Vinod Kumar/2011	Japan	HCV-infected patients	positive	negative	negative	HCC	non-HCV control	1394/7217	blood samples	multiplex PCR-based Invader assay and the Illumina HumanHap610-Quad	Yes	9
Vinod Kumar/2012	Japan	HBV-infected patients	negative	positive	negative	HCC	CHB, non-HBV control	407/6356	blood samples	Illumina Human Hap610-Quad and Human Hap550v3/Invader assay system	Yes	9
Giuseppa Augello/2018	Italy	HCV-infected patients	positive	negative	negative	HCC	LC healthy	150/335	blood samples	Competitive Allele-Specific KASP™ SNP genotyping platform	Yes	8
Christian M. Lange/2013	Switzerland	HCV-infected patients	positive	negative	negative	HCC	CHC	64/1860	blood samples	fluorescent-based competitive allele-specific PCR genotyping system	Yes	7

### Meta-analysis

**Fig. 2:**
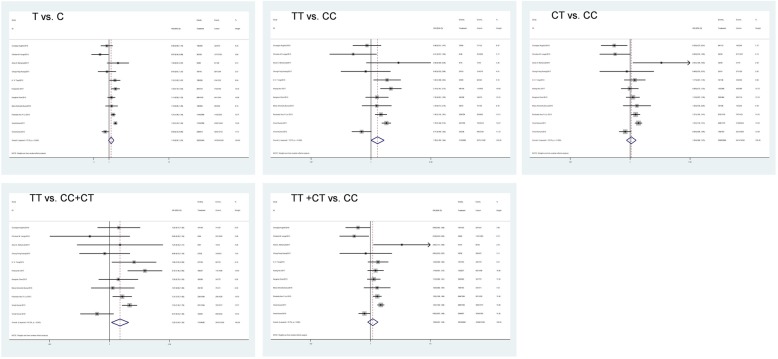
Forest plots of rs2596542 and HCC in different genetic models

Our analyses demonstrated heterogeneity among the results generated using the five genetic models [I^2^ > 50%, *P* (heterogeneity) < 0.10] (Table 3). Therefore, the results were pooled under a random effects model, and the results of the analysis showed that, under the recessive model, rs2569542 TT was a risk factor for the development of HBV/HCV-induced HCC. Individuals carrying the TT genotype were more genetically susceptible to HCC compared with other individuals [TT vs. CC/CT: OR = 1.248 (95% CI: 1.040–1.499), *P* = 0.017] (Table 3, Fig. 2).

**Table 3:** Combined results of genotype frequencies in HCC and non-HCC groups in different genetic models

Exposure	No-exposure	OR(95% CI)	*P* (OR)	I-squared	*P* (Heterogeneity)	*P* (Begg’s test)	*P* (Egger’s test)
T	C	1.102 (0.974, 1.248)	0.124	77.2%	0.000	0.533	0.156
CT	CC	1.041 (0.882, 1.230)	0.634	70.3%	0.000	0.876	0.247
TT	CC	1.279 (1.000, 1636)	0.050	72.7%	0.000	0.436	0.191
TT	CT/CC	1.248 (1.040, 1.499)	0.017	60.3%	0.005	0.350	0.194
TT/CT	CC	1.086 (0.911, 1.295)	0.356	76.7%	0.000	0.640	0.253

### Subgroup analyses

Subgroup analyses were conducted under the recessive model, according to control type, virus infection, and ethnicity. Patients with the TT genotype had higher susceptibility to HCC, regardless of whether they were HCV-infected (OR = 1.326, 95% CI: 1.101–1.599, *P* = 0.003), Asian (OR = 1.273, 95% CI: 1.002–1.618, *P* = 0.048), or were compared to a CHC control population (OR = 1.506, 95% CI: 1.172–1.936, *P* = 0.001) (Table 4, Additional file 1: Figure S1).

**Table 4:** Subgroup analysis results under recessive model

Group	Subgroup	No. of study	HBV/HCV patient	OR(95% CI)	*P* (OR)	I-squared	*P* (Heterogeneity)
Controls	LC	5	489	1.216 (0.984, 1.502)	0.070	0.0%	0.457
	CHC	4	1179	1.506 (1.172, 1.936)	0.001	58.2%	0.067
	CHB	3	243	1.218 (0.743. 1.997)	0.434	66.1%	0.052
	Healthy	5	1659	1.255 (0.899, 1.750)	0.182	68.6%	0.013
Population-based	HCV-infected patients	8	2452	1.326 (1.101, 1.599)	0.003	49.1%	0.056
	HBV-infected patients	3	936	1.133 (0.718, 1.788)	0.591	72.5%	0.026
Ethnicity	Asian	7	3135	1.273 (1.002, 1.618)	0.048	74.0%	0.001
	European	3	3613	1.065 (0.787, 1.440)	0.684	0.0%	0.455

### Publication bias

**Fig. 3:**
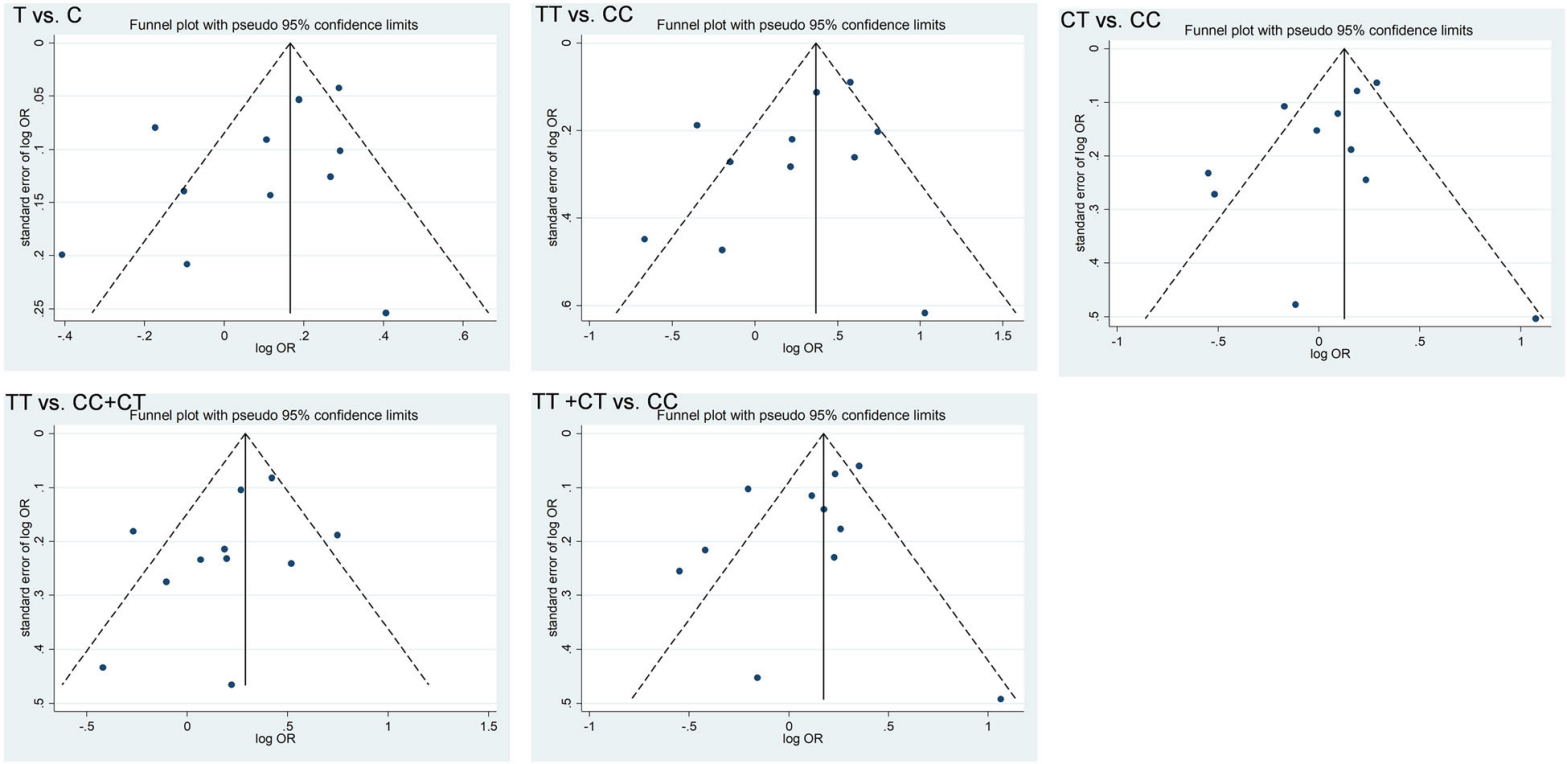
Funnel plots of Begg’s test to evaluate publication bias in the included studies

Publication bias was analyzed under the different genetic models, using Begg’s test to plot funnel diagrams. Ten studies among the 11 included articles are presented in the plots, and their data points were scattered and distributed, with the pooled OR value at the center; that is, they basically formed a symmetrical, inverted funnel-shape (Fig. 3). Analysis using Egger’s test demonstrated that all *P* values were > 0.05, suggesting no publication bias (Table 3).

## Discussion

This study systematically evaluated the relationship between rs2596542, in the *MICA* gene promoter, and HCC susceptibility. The results suggest that HBV/HCV-infected individuals carrying the TT genotype at this locus are more likely to develop HCC than those carrying other genotypes, particularly if they are HCV-infected or of Asian ethnicity. This may be due to the persistent HBV/HCV infection associated with the TT genotype [27, 31]. Further, infection with HBV/HCV can cause up-regulation of matrix metalloproteins, high levels of which lead to increased production of sMICA and decreased membrane-bound MICA tumor antigen protein [32, 33]. These changes ultimately inhibit the anti-tumor effects of immune cells and facilitate the immune escape of HCC cells [10, 12].

Compared with two previously published articles [24, 34], this study explores the relationship between rs2596542 and susceptibility to HBV/HCV-induced HCC. Only studies including cases of HBV/HCV-induced HCC were included in this meta-analysis, which used differing raw data and generated different results from previous studies. Among patients with persistent HCV infection, those with the TT genotype at rs2569542 are more likely to develop HCC, which is consistent with results of studies in Asian populations, suggesting that the susceptibility of HBV/HCV-induced HCC is not only related to individual genetic factors, but also to viral infection and geographical region. Heterogeneity remained after subgroup analysis, which may be because we did not consider the effect of viral genotype, sex, or complications on the influence of genotype on HCC risk.

In this study, a qualitative Begg’s test plot and quantitative Egger’s test analysis showed no significant publication bias, indicating that the combined results are reliable; however, the study had some limitations. First, the publications included in this study were limited to texts published in English, hence publication and cultural bias may still have affected the results of the meta-analysis. Second, the majority of genotype data in this study were derived from Chinese and Japanese individuals; this lack of genotype data from other ethnic groups reduces the wider applicability of our findings.

## Conclusion

In summary, rs2596542 is a molecular marker for HCC, and HCV/HBV-infected individuals carrying the TT genotype of this SNP have higher genetic susceptibility to HCC than HCV/HBV-infected individuals carrying the CT/CC genotypes. In particular, among HCV-infected populations and Asians, individuals carrying the TT genotype are more likely to develop HCC than those carrying CC/CT genotypes. This study provides a theoretical basis for the personalized treatment of virus-infected individuals with HCC.

## Additional file

Additional file 1: **Figure S1.** Forest plots of rs2596542 and HCC for subgroup analyses. (JPG 1689 kb)

## Data Availability

All data generated or analyzed during this study are included in this manuscript.

## Abbreviations

CHB: Chronic hepatitis B
CHC: Chronic hepatitis C
CI: Confidence interval
HBV: Hepatitis B virus MICA Major histocompatibility complex class I-related gene A
HCC: Hepatocellular carcinoma
HCV: Hepatitis C virus
HWE: Hardy-Weinberg equilibrium
LC: Live cirrhosis
OR: Odds ratio
sMICA: Soluble MICA
SNP: Single nucleotide polymorphism

## References

[1] Aguilar-Olivos N, Ornelas-Arroyo S, Chavez-Tapia NC, Uribe M, Méndez-Sánchez N (2013). New insights in the diagnosis, pathogenesis and treatment of hepatitis B-and C-related hepatocellular carcinoma. Curr Hepat Rep.

[2] Sangiovanni A, Prati GM, Fasani P, Ronchi G, Romeo R, Manini M, Del Ninno E, Morabito A, Colombo M (2006). The natural history of compensated cirrhosis due to hepatitis C virus: a 17-year cohort study of 214 patients. Hepatology.

[3] El-Serag HB (2012). Epidemiology of viral hepatitis and hepatocellular carcinoma. Gastroenterology.

[4] Huang CF, Yeh ML, Tsai PC, Hsieh MH, Yang HL, Hsieh MY, Yang JF, Lin ZY, Chen SC, Wang LY (2014). Baseline gamma-glutamyl transferase levels strongly correlate with hepatocellular carcinoma development in non-cirrhotic patients with successful hepatitis C virus eradication. J Hepatol.

[5] El-Serag HB (2011). Hepatocellular carcinoma. N Engl J Med.

[6] Llovet JM, Burroughs A, Bruix J (2003). Hepatocellular carcinoma. Lancet.

[7] Nguyen VT, Law MG, Dore GJ (2009). Hepatitis B-related hepatocellular carcinoma: epidemiological characteristics and disease burden. J Viral Hepat.

[8] Sarma MP, Asim M, Medhi S, Bharathi T, Diwan R, Kar P (2012). Viral genotypes and associated risk factors of hepatocellular carcinoma in India. Cancer Biol Med.

[9] Bahram S, Bresnahan M, Geraghty DE, Spies T (1994). A second lineage of mammalian major histocompatibility complex class I genes. Proc Natl Acad Sci.

[10] Bauer S, Groh V, Wu J, Steinle A, Phillips JH, Lanier LL, Spies T (1999). Activation of NK cells and T cells by NKG2D, a receptor for stress-inducible MICA. Science.

[11] Diefenbach A, Jensen ER, Jamieson AM, Raulet DH (2001). Rae1 and H60 ligands of the NKG2D receptor stimulate tumour immunity. Nature.

[12] Groh V, Wu J, Yee C, Spies T (2002). Tumour-derived soluble MIC ligands impair expression of NKG2D and T-cell activation. Nature.

[13] Chen D, Gyllensten U (2014). MICA polymorphism: biology and importance in cancer. Carcinogenesis.

[14] Wang Y, Zhang N, Chen E, Chen C, Bu Y, Yu P (2016). Allele polymorphism and haplotype diversity of MICA/B in Tujia nationality of Zhangjiajie, Hunan Province, China. Hum Immunol.

[15] Kumar V, Kato N, Urabe Y, Takahashi A, Muroyama R, Hosono N, Otsuka M, Tateishi R, Omata M, Nakagawa H (2011). Genome-wide association study identifies a susceptibility locus for HCV-induced hepatocellular carcinoma. Nat Genet.

[16] Augello G, Balasus D, Fusilli C, Mazza T, Emma MR, Giannitrapani L, Agliastro R, Cervello M, Montalto G (2018). Association between MICA gene variants and the risk of hepatitis C virus-induced hepatocellular Cancer in a Sicilian population sample. OMICS.

[17] Huang CF, Huang CY, Yeh ML, Wang SC, Chen KY, Ko YM, Lin CC, Tsai YS, Tsai PC, Lin ZY (2017). Genetics variants and serum levels of MHC class I chain-related a in predicting hepatocellular carcinoma development in chronic hepatitis C patients post antiviral treatment. EBioMedicine.

[18] Lange CM, Bibert S, Dufour JF, Cellerai C, Cerny A, Heim MH, Kaiser L, Malinverni R, Mullhaupt B, Negro F (2013). Comparative genetic analyses point to HCP5 as susceptibility locus for HCV-associated hepatocellular carcinoma. J Hepatol.

[19] Guo J, Jin M, Zhang M, Chen K (2012). A genetic variant in miR-196a2 increased digestive system cancer risks: a meta-analysis of 15 case-control studies. PLoS One.

[20] Li K, Tie H, Hu N, Chen H, Yin X, Peng C, Wan J, Huang W (2014). Association of two polymorphisms rs2910164 in miRNA-146a and rs3746444 in miRNA-499 with rheumatoid arthritis: a meta-analysis. Hum Immunol.

[21] Thakkinstian A, McEvoy M, Minelli C, Gibson P, Hancox B, Duffy D, Thompson J, Hall I, Kaufman J, Leung TF (2005). Systematic review and meta-analysis of the association between β2-adrenoceptor polymorphisms and asthma: a HuGE review. Am J Epidemiol.

[22] Camargo MC, Mera R, Correa P, Peek RM, Fontham ET, Goodman KJ, Piazuelo MB, Sicinschi L, Zabaleta J, Schneider BG (2006). Interleukin-1β and interleukin-1 receptor antagonist gene polymorphisms and gastric cancer: a meta-analysis. Cancer Epidemiol Prev Biomarkers.

[23] Gao LB, Pan XM, Li LJ, Liang WB, Zhu Y, Zhang LS, Wei YG, Tang M, Zhang L (2011). RAD51 135G/C polymorphism and breast cancer risk: a meta-analysis from 21 studies. Breast Cancer Res Treat.

[24] Kuang XJ, Mo DC, Qin Y, Ahir BK, Wang JJ, Peng Z, Deng ZL (2019). Single nucleotide polymorphism of rs2596542 and the risk of hepatocellular carcinoma development: a meta-analysis. Medicine (Baltimore).

[25] Mohamed AA, Elsaid OM, Amer EA, Elosaily HH, Sleem MI, Gerges SS, Saleh MA, El Shimy A, El Abd YS (2017). Clinical significance of SNP (rs2596542) in histocompatibility complex class I-related gene a promoter region among hepatitis C virus related hepatocellular carcinoma cases. J Adv Res.

[26] Hai H, Tamori A, Yoshida K, Hagihara A, Kawamura E, Uchida-Kobayashi S, Morikawa H, Enomoto M, Murakami Y, Kawada N (2017). Polymorphisms in MICA, but not in DEPDC5, HCP5 or PNPLA3, are associated with chronic hepatitis C-related hepatocellular carcinoma. Sci Rep.

[27] Chen K, Shi W, Xin Z, Wang H, Zhu X, Wu X, Li Z, Li H, Liu Y (2013). Replication of genome wide association studies on hepatocellular carcinoma susceptibility loci in a Chinese population. PLoS One.

[28] Burza MA, Motta BM, Mancina RM, Pingitore P, Pirazzi C, Lepore SM, Spagnuolo R, Doldo P, Russo C, Lazzaro V (2016). DEPDC5 variants increase fibrosis progression in Europeans with chronic hepatitis C virus infection. Hepatology.

[29] Lo PHY, Urabe Y, Kumar V, Tanikawa C, Koike K, Kato N, Miki D, Chayama K, Kubo M, Nakamura Y (2013). Identification of a functional variant in the MICA promoter which regulates MICA expression and increases HCV-related hepatocellular carcinoma risk. PLoS One.

[30] Kumar V, Lo PHY, Sawai H, Kato N, Takahashi A, Deng Z, Urabe Y, Mbarek H, Tokunaga K, Tanaka Y (2012). Soluble MICA and a MICA variation as possible prognostic biomarkers for HBV-induced hepatocellular carcinoma. PLoS One.

[31] Tong HV, Toan NL, Song LH, Bock CT, Kremsner PG, Velavan TP (2013). Hepatitis B virus-induced hepatocellular carcinoma: functional roles of MICA variants. J Viral Hepat.

[32] Lara-Pezzi E, Gomez-Gaviro MV, Galvez BG, Mira E, Iniguez MA, Fresno M, Martinez AC, Arroyo AG, Lopez-Cabrera M (2002). The hepatitis B virus X protein promotes tumor cell invasion by inducing membrane-type matrix metalloproteinase-1 and cyclooxygenase-2 expression. J Clin Invest.

[33] Salih HR, Rammensee HG, Steinle A (2002). Cutting edge: down-regulation of MICA on human tumors by proteolytic shedding. J Immunol.

[34] Tu C, Chen W, Wang S, Tan W, Guo J, Shao C, Wang W. MicroRNA-383 inhibits doxorubicin resistance in hepatocellular carcinoma by targeting eukaryotic translation initiation factor 5A2. J Cell Mol Med. 2019. 10.1111/jcmm.14197. 10.1111/jcmm.14197 PMC6815770 30801960

