# Supplementary figures and images for: Relationship between the rs2596542 polymorphism in the MICA gene promoter and HBV/HCV infection-induced hepatocellular carcinoma: a meta-analysis
Source: BMC Med Genet. 2019 Aug 16;20:142. doi: 10.1186/s12881-019-0871-2 (PMC6697945; doi:10.1186/s12881-019-0871-2)

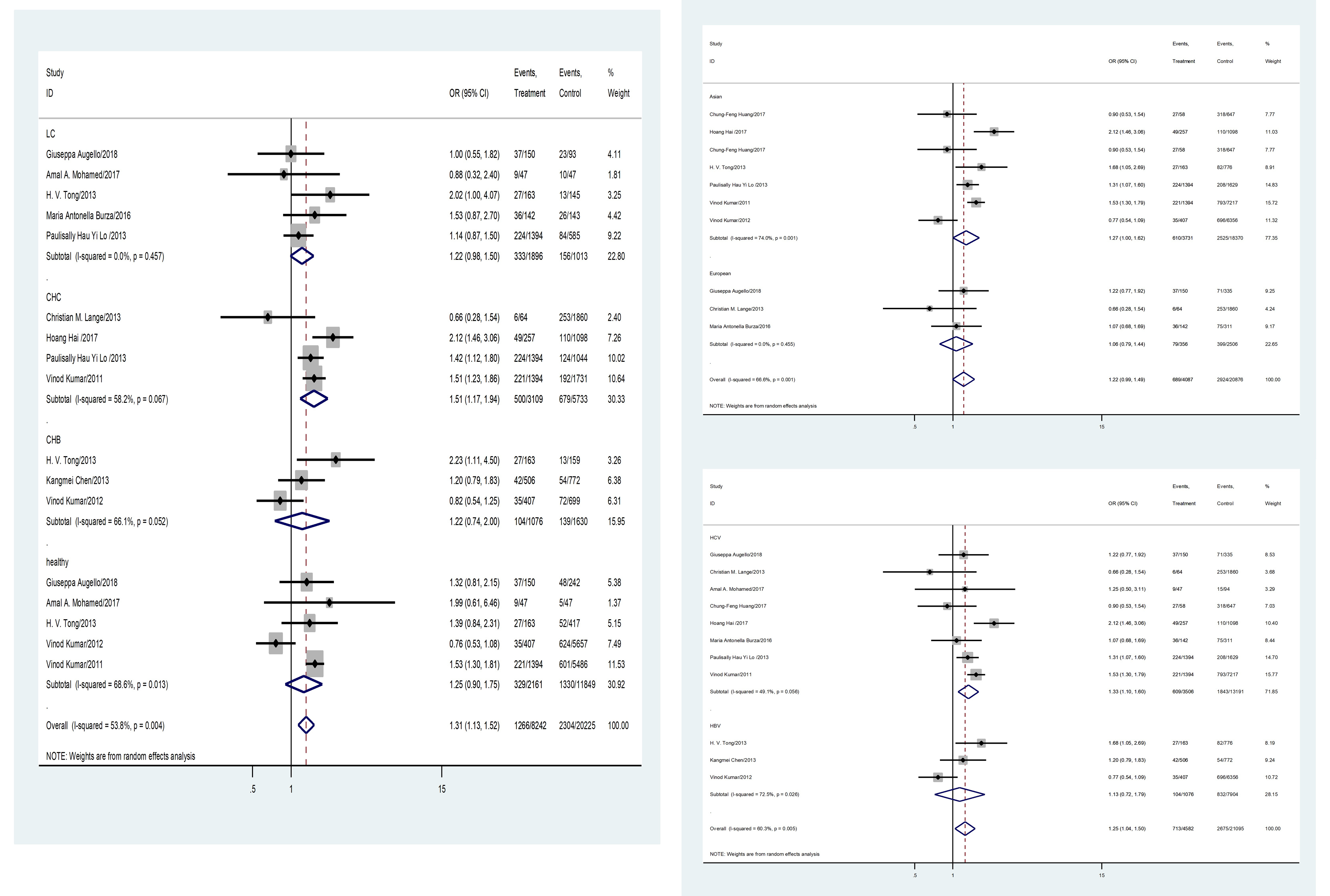

Supplement: Supplementary file 1 — Figure S1. Forest plots of rs2596542 and HCC for subgroup analyses. (JPG 1689 kb) [file 12881_2019_871_MOESM1_ESM.jpg]
